# A systematic review of US state environmental legislation and regulation with regards to the prevention of neurodevelopmental disabilities and asthma

**DOI:** 10.1186/1476-069X-8-9

**Published:** 2009-03-26

**Authors:** Lauren Zajac, Eli Sprecher, Philip J Landrigan, Leonardo Trasande

**Affiliations:** 1 Department of Community and Preventive Medicine, Mount Sinai School of Medicine, New York, NY, USA; 2 Department of Pediatrics, Mount Sinai School of Medicine, New York, NY, USA

## Abstract

**Background:**

While much attention is focused on national policies intended to protect human health from environmental hazards, states can also prevent environmentally mediated disease through legislation and regulation. However, relatively few analyses have examined the extent to which states protect children from chemical factors in the environment.

**Methods:**

Using Lexis Nexis and other secondary sources, we systematically reviewed environmental regulation and legislation in the fifty states and the District of Columbia as of July 2007 intended to protect children against neurodevelopmental disabilities and asthma.

**Results:**

States rarely address children specifically in environmental regulation and legislation, though many state regulations go far to limit children's exposures to environmental hazards. Northeast and Midwest states have implemented model regulation of mercury emissions, and regulations in five states set exposure limits to volatile organic compound emissions that are more stringent than US Environmental Protection Agency standards.

**Discussion:**

Differences in state environmental regulation and legislation are likely to lead to differences in exposure, and thus to impacts on children's health. The need for further study should not inhibit other states and the federal government from pursuing the model regulation and legislation we identified to prevent diseases of environmental origin in children.

## Introduction

More than 80,000 new synthetic chemicals have been developed and disseminated in the United States over the past 50 years. Children are at special risk of exposure to the 2,800 high-volume chemicals that are produced in quantities greater than one million tons per year and that are most widely dispersed in air, water, food crops, communities, waste sites and homes.[1] Rates of many common diseases are increasing in American children, and evidence is accumulating that environmental exposures are partially responsible for these alarming trends.[2] These illnesses include asthma,[3,4] certain childhood cancers,[5,6] certain birth defects, [7-9] and neurodevelopmental disabilities. [10-12]

Federal regulation of chemicals that are widely dispersed into the environment through industrial and other activities has proven successful in the reduction of childhood disease and disability.[13] Reductions in exposure associated with the elimination from lead in gasoline in the United States resulted in IQs among preschool aged children in the 1990s that were 2.2–4.7 points higher than they would have been if those children had a distribution of blood lead levels found among children in the 1970s [14]. Before the US Environmental Protection Agency's (USEPA) phase out of diazinon and chlorpyrifos, these two pesticides were frequently detected in the cord blood of New York City children and associated with decrements in birth weight and length. After these phaseouts, the pesticides and the association with predictors of cognitive potential were no longer detected [15]. Local policy can also dramatically influence children's exposure to environmental chemicals, and result in reductions in childhood morbidity.

Restrictions instituted by the city of Atlanta on vehicular travel during the 2000 Olympic Games were associated with significant reductions in ambient ozone and in asthma acute care events [16].

States also can influence the environment in which children live, work (i.e., go to school), and play, both through public health programs and policies as well as environmental regulation and legislation. Public health programs and policies, such as fish advisories for mercury, can reduce exposure to chemical factors in the environment, [17] but they are routinely implemented under broad public health authority, and no database exists to analyze how states implement programs to reduce chemical exposures under this authority.

National and international agencies have developed reports to evaluate the effectiveness of environmental legislation and regulation designed to protect children from environmental hazards. The World Health Organization (WHO) has developed children's environmental health indicators so that countries can judge the effectiveness of their legislation and regulation. [18] USEPA has produced reports on America's Children and the Environment which utilize data from extant national sources to evaluate trends in children's exposure to environmental chemicals and diseases of environmental origin over time [19].

State regulation and legislation can serve as models for the improvement of national policy, yet few comparisons of state policy have been made. The National Conference of State Legislatures has produced a database of proposed and enacted legislation on issues of children's environmental health, but does not compare the effectiveness of state approaches to protecting children from environmental hazards [20]. We therefore chose to review systematically environmental regulation and legislation that has been enacted in the fifty states with respect to children's environmental health. A major goal of our analysis was also to identify initiatives that could serve as models for disease prevention in other states and across the country. We focused our review upon environmental regulation and legislation with regard to prevention in two disease categories: neurodevelopmental disabilities and asthma, those for whom the most evidence has been established for causation of environmental factors, and for possible prevention through environmental regulation and legislation. [13]

## Methods

We began by selecting chemicals which had sufficiently strong evidence to support a possible etiologic link with neurodevelopmental disabilities and asthma. In this effort, the authors relied heavily upon a recent systematic review of the industrial chemicals known to be neurotoxic in humans, [21] and the American Academy of Pediatrics (AAP) text Pediatric Environmental Health. [22] The AAP text was the authors' main source for identifying chemicals which had sufficiently strong evidence to support a possible epidemiologic association with the exacerbation or development of asthma. This approach yielded the following list of candidate chemicals for neurodevelopmental disabilities: pesticides, mercury, polychlorinated biphenyls (PCBs), arsenic, dioxins, lead, and environmental tobacco smoke. We chose not to include perfluorinated compounds, polybrominated diphenylethers (PBDEs), manganese, perchlorate and flouride due to the lack of high quality human studies. [21] For asthma, the authors identified mold, volatile organic compounds, diesel exhaust, criteria air pollutants and environmental tobacco smoke as chemical factors with sufficient evidence for further analysis. As a secondary confirmation, we confirmed that these chemicals have been associated with impacts on neurodevelopment or asthma in one or more human studies.[15,23-32] A lack of human studies prevented us from examining the impact of mixtures in assessing candidate chemicals in this study.

While "best practices" do exist for clinical and public health practice,[33,34] few studies have compared the effectiveness of state-level regulatory/legislative interventions to reduce exposure. [13] As the WHO indicators for children's environmental health are currently only designed to judge legislative and regulatory success,[18] we could not use the WHO criteria to judge whether legislative or regulatory approaches were effective. We therefore relied upon knowledge of the exposure pathways, bioavailability and toxicity for each of these chemicals to judge regulation and legislation in different states with respect to the extent to which they could reduce burden of disease. [13] Policies are identified in this manuscript if, based upon a review of the literature cited in this manuscript, they were judged by two of the authors to be able to reduce exposure to one of the chemicals that have been associated with neurodevelopmental disabilities or asthma. Those legislative and regulatory efforts that were judged by two of the authors to reduce exposure most effectively when fully implemented were identified as "model legislation" and "model regulation." A summary of the criteria used for each chemical exposure in judging legislation and regulation is provided below.

For lead exposure, we identified lead-based paint hazards as the major source of childhood lead exposure, and focused our analysis on primary prevention (e.g., efforts to encourage eradication of hazards) and secondary prevention efforts (e.g., screening programs), recognizing that the former programs have been cited as the most effective programs to produce reductions in children with lead poisoning. [26] While use of lead paint in toys does appear to pose a hazard, this was deemed a lower risk for exposure across the population of children, and regulation and legislation that banned lead in toys were not considered model policies for prevention. For mercury exposure, for example, the authors focused their analysis on policies that limited exposure to methylmercury, as ethylmercury exposure has not been associated with adverse effects on neurodevelopment in multiple human studies.[35] Consumption of contaminated fish is the major source of human exposure to methylmercury, and the authors used Toxic Release Inventory data to identify most common sources of methylmercury contamination through mercury emissions, such as coal-fired power plants. [36,37]

For pesticide exposures, the authors identified three major pathways – take-home exposures for children with parents who work in agricultural settings, [38] residues from food sprayed with pesticides,[39] and home exposures when pesticides are sprayed to treat infestations.[40] Recognizing that these exposure pathways contribute to different degrees in individual children, the authors chose to weight each pathway equally in considering legislation and regulation. For arsenic, water contamination [41] and dermal exposure through play on copper chromium arsenate wood [42] were considered the most effective pathways, while soil contamination was identified as another contributor. [43] Human milk exposure, the primary pathway for exposure to dioxin, [22] was deemed unlikely to be immediately reduced through environmental regulation and legislation, and fish advisories were identified to be largely executed through broad public health authority. While dermal, ingestion and inhalational exposures to dioxin are likely to contribute less to children's daily exposure, we did compare states with respect to the regulation and legislation they have implemented to reduce exposures through these pathways.

Exposures to criteria air pollutants are largely a product of safety thresholds set by USEPA and federal legislation such as the Clean Air Act,[29] but we considered state efforts to supersede these thresholds and to encourage clean fuel usage. We also considered school bus idling and retrofit programs for school buses [44] given that children spend five days per week and nine months per year commuting to and from school. We also identified as models those regulations that could reduce exposure to volatile organic compounds (VOC) and mold in schools given the many hours that young children spend in these environments, and the evidence that these exposures can contribute to asthma exacerbations. [45,46] Building materials are more likely to contribute to VOC emissions in the long-term than furnishings or consumer products, [22] and we therefore we considered as models those laws and regulations that could effectively limit these exposures. Programs to eradicate mold exposure in homes were identified as being implemented under broad public health authority, and therefore not included in this analysis.

We then proceeded to develop a search method for these chemicals to identify the relevant environmental regulation and legislation in each of the fifty states and the District of Columbia. Lexis-Nexis^® ^(LN) is internationally known as the leading database for legal documents, and provides ongoing updates of legislative and regulatory activity in each of the fifty states. The State Codes, Constitutions, Court Rules & Advance Legislative Service combined group file [47] contains the statutory codes, state constitutions, court rules and current laws from the fifty states. We began our analysis by running preliminary searches with each of the chemicals we identified in LN and made further refinements in the search terms to ensure that our search terms did not inadvertently exclude results on the basis of differing terminology or nomenclature. Table 1 presents the chemicals and the associated search terms we used for neurodevelopmental disabilities and asthma.

**Table 1:** Search Terms Applied in the Systematic Review

Toxin	Search Terms (Lexis-Nexis Unless Otherwise Noted)
Pesticides	Heading "pesticide" AND "Integrated Pest Management" OR "integrated w/ pest w/ management" Heading "pesticide" OR "pest" AND (full-text) "schools" or "daycare" "pesticide" AND "notification" OR "notice" OR "notify" "pesticide" in heading AND "exposure" AND "occupational" OR "worker" OR "employee"

Mercury	"mercury" AND "emissions" "mercury" AND "incinerate" OR "incineration" OR "dispose" OR "disposal" "mercury" AND "thermometers" OR "devices" OR "esophageal dilators, bougie tubes, gastrointestinal tubes" OR "blood pressure" OR "sphygmomanometers"

Polychlorinated Biphenyls (PCBs)	"Polychlorinated Biphenyl" OR "PCBs" AND "waste" OR "disposal" OR "dispose"

Dioxins	"dioxin"

Arsenic	"Arsenic" AND "water" "Copper Chromium Arsenate" AND "wood" "Pressure Treated Wood" AND "Arsenic" "Wood" AND "Arsenic" "Arsenic" AND "playground" "Arsenic" AND "maximum contaminant level"

Lead	Heading "Lead" AND "screening" Heading "Lead" AND "report" OR "reporting" OR "tracking" Heading "Lead" AND "tax credit" OR "loan" Heading "Lead" AND "prevent" OR "prevention" OR "education" OR "educate" Heading "Lead" AND "paint"

Environmental Tobacco Smoke	Search terms from American Lung Association's State Legislated Actions on Tobacco Issues (SLATI) Database at http://slati.lungusa.org/ State Regulatory Agency websites

Healthy Homes and Schools: Mold, Volatile Organic Compounds, and other Indoor Air Pollution	"indoor air quality" "Volatile Organic Compounds" California Air Resources Board search for consumer products "mold" AND "indoor" OR "home" "green cleaning" OR "environmentally-sensitive cleaning" OR ("green" AND "cleaning" OR "clean" OR "supplies") Secondary Source for Executive Orders: DSIRE (Database of State Incentives for Renewables & Efficiency) "Energy Standards for Public Buildings for Energy Efficiency" http://www.dsireusa.org

Diesel Exhaust	"School bus" AND "emissions" Secondary Sources: Environmental Protection Agency, "Compilation of State, County, and Local Anti-Idling Regulations," April 2006, http://www.epa.gov/smartway/documents/420b06004.pdf and Union of Concerned Scientists "School Bus Pollution Report Card 2006," http://www.ucsusa.org/assets/documents/clean_vehicles/pollution-report-card-2006.pdf

Toxin	Search Terms (Lexis-Nexis Unless Otherwise Noted)

Criteria Air Pollutants and the National Ambient Air Quality Standards	USEPA Green Book http://www.epa.gov/air/oaqps/greenbk/

Volatile Organic Compounds	"Volatile Organic Compounds" State Environmental Regulatory Agency regulation searches of states in the Ozone Transport Commission (Connecticut, Delaware, the District of Columbia, Maine, Maryland, Massachusetts, New Hampshire, New Jersey, New York, Pennsylvania, Rhode Island, Vermont, and Virginia)

When LN search results or secondary sources (see Table 1) referred to a specific state regulation, we subsequently obtained the regulation from the relevant state agency's website, and reviewed the entire statute for completeness. When our search results referred to another statute, we went directly to this statute and determined relevancy for the report. The relevant results (as of July 2007) were summarized for each chemical and policies were organized into issue area subgroups. For example, we divided the diesel exhaust legislation results into three distinct subgroups: diesel emissions, school bus policies, and diesel truck and commercial vehicle idling.

As we could not identify a previous published approach to analyzing state environmental regulation and legislation with regard to children's environmental health, we performed a final validity screen to our results. We compared our results using LN and secondary sources with those from the Centers for Disease Control and Prevention's Childhood Lead Poisoning Prevention Program's review of state programs for lead, [48] the American Lung Association's review of state tobacco regulation and legislation, [49] and the review performed by National Conference of State Legislatures for other chemical exposures.[50]

Having divided the results of our search into issue area subgroups, two of the authors assessed whether the legislation/regulation could actually reduce children's exposure. Two of the authors then compared effectiveness of the regulatory/legislative initiatives, and identified the regulatory/legislative initiatives that could most effectively reduce exposure. These initiatives were identified as "models" for other states to consider. The results presented below represent a comparison of states with respect to approaches they have taken to protect children from neurodevelopmental disabilities and asthma, and an identification of those enacted laws and regulations that are most likely to prevent disease and disability from environmental exposures. We identify these laws and regulations as "model legislation" and "model regulation" to call attention to them in the subsequent section.

## Results

### Neurodevelopmental Disabilities

With the exception of lead, state environmental regulation and legislation rarely addressed children specifically. However, states did vary in the number of approaches that were intended to protect the general public and effective in limiting morbidity from neurotoxic exposures.

**Figure 1:**
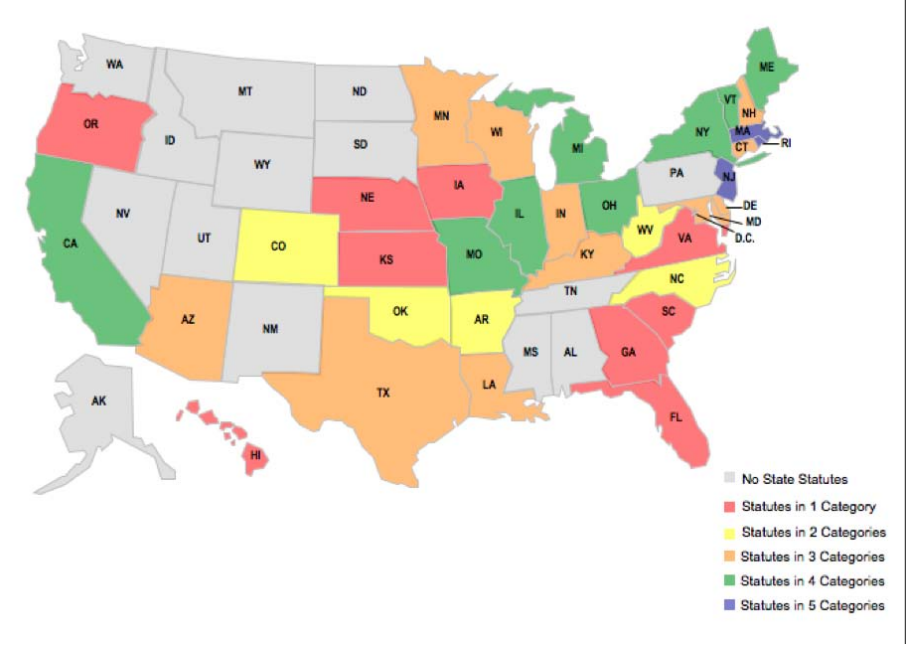
**Comparison of State Lead Regulation/Legislation**.

In the five categories of lead paint that we examined (screening, data tracking, lead abatement funding, lead prevention programs, and lead paint bans), eleven states had regulation or legislation in four or more areas, twenty-eight had in two or more, and thirty-six (and the District of Columbia) had policies in at least one area (Figure 1). We identified only thirty states which statutorily require screening, but also found that nearly all states have established a screening program through broad public health authority. [48] This was the only area in which our validity screen yielded different results from that which we obtained using our base methodology. Many states exceeded the Centers for Disease Control and Prevention guidelines that encourage screening of high risk children by requiring mandatory screening of all children. Massachusetts has model regulation that most proactively identifies children with lead poisoning by requiring the most intensive screening regimen, with the first test done between the ages of 9 and 12 months, followed by testing at ages 2 and 3. Children in high risk areas must also be tested at age 4 years.

Most states statutorily require the tracking of elevated lead levels: some states require the reporting of all results, while others require the reporting only of elevated lead levels, often defined as ≤ 10 μg/dL. Statutes in California and New Jersey require that databases contain geographical data that can be used to map locations where lead poisoning has occurred, which permits further targeting for lead hazard abatement efforts and primary prevention. A number of states also have implemented legislation that is likely to be extremely effective in preventing childhood lead poisoning, ranging from tax credits to grants and loans for lead abatement (Rhode Island, Massachusetts, Missouri and Minnesota). Many states have limited or banned the use of lead paint in common products that are accessible to children, such as toys, but these efforts are less likely to reduce the burden of lead poisoning than programs to eliminate lead-based paint hazards in homes.

**Figure 2:**
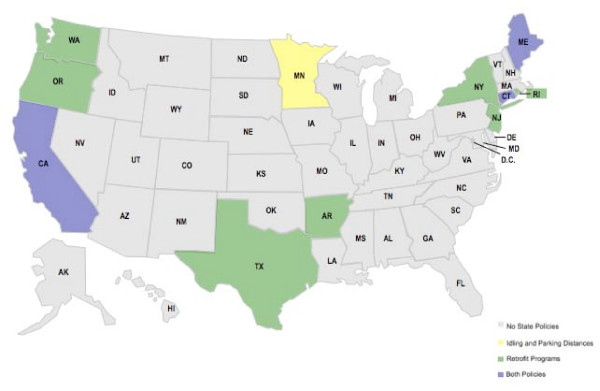
**Comparison of State Mercury Regulation/Legislation**.

In limiting mercury exposure, multiple states in the Northeast and Midwest have instituted model legislation to prevent prenatal methylmercury toxicity (Figure 2). Connecticut, New Hampshire, Maryland, Michigan and Pennsylvania have implemented model regulations that protect children from this neurotoxic exposure by lowering mercury emissions from coal-fired power plants to 80–90% of 1990 levels. While USEPA has banned use and manufacture of polychlorinated biphenyls (PCBs), another fish contaminant that has been documented to cause damage to the developing brain, twenty-eight states also have statutes regarding its disposal. California bans the incineration of PCB containing materials while Connecticut, Idaho, Indiana, Minnesota and North Carolina limit it or have notification requirements for PCB incineration.

Twenty-six states and the District of Columbia have enacted model legislation that bans smoking in all, or virtually all, public places such as bars, restaurants, places of employment, schools and child-care centers, and three more states ban smoking in most public places. Of the twenty-one states that have not enacted wide-ranging indoor-smoking bans, virtually all of them ban smoking in schools, daycare and healthcare facilities, although some states permit smoking in designated areas.

While twelve states require Integrated Pest Management (IPM) for school grounds and/or public lands, only California, Texas, and West Virginia require the use of least toxic pesticides, which is most likely to reduce risk for neurodevelopmental impact. California also has enacted legislation that requires notification for pesticide spraying in other public facilities such as parks, going so far as to requiring the posting of bilingual signs. Maine and New Hampshire require public notification for aerial spraying. These laws empower parents somewhat to prevent exposure, and should be considered as a model approach for other states to prevent disease, but not a very good model in that it gives the citizen a "choice" to essentially restrict his/her activities or those of his/her family in order to accommodate the application of pesticides. More effective approaches would limit use of pesticides in public places to the least toxic available or eliminate pesticide use entirely. California is the only state to ban pharmaceutical use of Lindane, an organochloride pesticide used to treat lice and scabies.

A number of states have enacted other pesticide regulations that are less likely to reduce children's exposure to pesticides. Louisiana and Pennsylvania require schools to maintain a pesticide sensitivity registry, and Michigan, Colorado and Washington maintain registries of patients who are certified by a physician to be sensitive to pesticide use, so that they receive prior notification before pesticide use in their area. These registries unfortunately do not fully recognize the scientific knowledge that pesticides can cause toxicity to the developing brain in populations who are not classified as sensitive. New York has a registry to track all pesticide use in the state, which may be useful to guide studies for possible etiologic associations, but have limited promise otherwise to prevent disease.

Recent federal activity has resulted in a lower maximum contaminant level for arsenic (10 parts per billion) in water and a voluntary withdrawal of arsenic-containing wood products from market. With the exception of California, which required a disclosure for bottled water with arsenic levels between 5 and 10 parts per billion in October 2007, states have thus far pursued few additional efforts besides funding programs to remove arsenic from water supplies (California and New Mexico). Through our systematic review, we identified no state with a lower maximum contaminant level than USEPA, even though toxicity has been documented at lower levels of exposure.[51] North Carolina has prohibited chromated copper arsenate-treated wood for future use on school grounds, and requires testing for arsenic in new private wells. No state has proceeded to implement regulation or legislation that bans the sale of chromated copper arsenate treated wood, though a plan has been announced by USEPA to have producers voluntarily cease sale of this wood.[52]

States have also undertaken a number of statutory efforts to limit dioxin contamination, monitor and limit its emissions, or outright ban it. While some states have instituted caps on dioxin emissions from waste treatment facilities and require testing of dioxin emissions, Maine, New Jersey and New Hampshire have model regulation that requires "best available" technology be used to limit dioxin emissions. Maine also has established a "Dioxin Monitoring Program" to test for levels near wastewater treatment plants and fish in their waters. New Hampshire has enacted model legislation that bans building of new medical waste incinerators and will prohibit all medical waste incineration, a major source of dioxin exposure, by 2014. A number of states have banned dioxin in dust-mitigating compounds and the District of Columbia and Georgia have banned the use of all dioxin containing materials for dust suppression or road treatment. Further, action levels for remediation vary widely across states. Oregon requires action at 3.9 parts per trillion (ppt), while Minnesota requires action at 200 ppt.

### Asthma

We identified few states that specifically designed regulation and legislation to protect children from asthma, but did identify a number of approaches that many states took to protect the whole population and also are effective in limiting morbidity from childhood asthma. In the previous section, we described differences in state legislation and regulation with regard to tobacco smoke, which is associated with impacts on neurodevelopment and asthma. California has consistently protected children by exceeding federal diesel emissions requirements, instituting a cap on particulate matter emissions beginning in model year 2004. Several years later, the federal government implemented similar regulations including requiring ultra-low sulfur diesel fuel and capping particulate matter emissions from heavy duty engines. Three other states have implemented additional diesel policies including emissions testing and purchasing of low emission vehicles for use by state agencies. In addition, fourteen states have implemented legislation or regulation that limits the time allowed for idling of diesel-fueled commercial vehicles.

**Figure 3:**
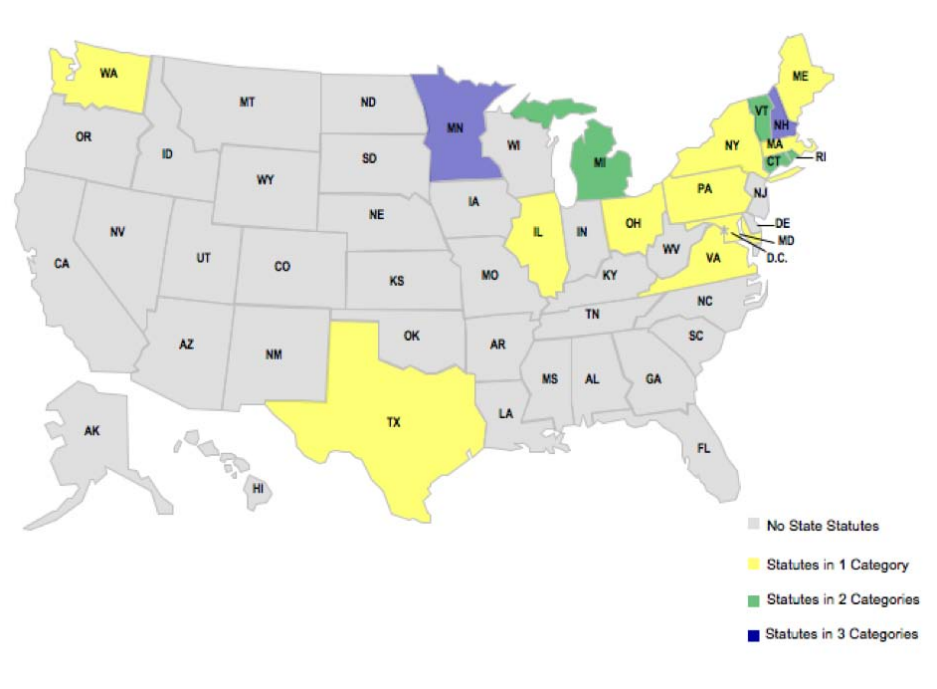
**School Bus Regulation/Legislation Among States**.

To limit exposures in the school setting, four states have implemented legislation or regulation that limits the time allowed for school bus idling and/or requires a minimum distance for the parking of buses near school buildings (Figure 3). However, Connecticut is the only state to explicitly enforce these limits and ensure that these regulations have their intended effect of protecting children by making it a finable infraction to leave a school bus idling for more than three consecutive minutes. Ten states have implemented retrofit programs for school buses, but only Rhode Island has required that these retrofit programs will be implemented by September 2010, thereby reducing exposures to children most immediately and effectively. Other state retrofitting programs have uncertain impact in reducing childhood exposures because they only provide inducements (e.g., grants) to encourage implementation. The Rhode Island legislation also requires that newer buses either be retrofitted with a crankcase ventilation system; a model year 2007 or later engine; or the use of alternative fuels, such as compressed natural gas, which achieve reductions of diesel particulate matter (DPM) emissions.

Illinois, Maine, and New York have passed legislation that most effectively reduces exposure to VOCs in school indoor air by requiring schools to phase in the use of green cleaning products. In contrast, Connecticut only requires air quality testing for compounds such as VOCs in school buildings built or renovated in 2003 or beyond, and Maryland only requires portable classrooms to be built with materials that have low VOC content. Several states including Connecticut, West Virginia, and Wisconsin have set specific indoor air quality standards for schools. Of the nine states have policies for indoor air quality monitoring and/or assessment for public and/or private dwellings, only five states have taken the most effective approach of establishing specific standards or guidelines for indoor air quality. Oregon is the only of these states that has enacted regulations that most effectively protect children by requiring an adequate margin of safety for sensitive populations. Nine states have included indoor air quality improvements in the definition of energy savings measures that are eligible for tax breaks or other financial incentives, or exempt from regulatory limitations. These fiscal incentives are also very effective approaches that other states should consider to encourage limitation of indoor air pollutants that increase morbidity from asthma.

Five states have more aggressive safety thresholds than USEPA for volatile organic compound (VOC) emissions from indoor materials or consumer products. Of these, California and Rhode Island have implemented model regulation that most proactively protects children by banning the sale or manufacture of products with VOC levels greater than state standards, while Maine has model regulation that bans the sale or manufacture of any "architectural or industrial maintenance coating," manufactured after January 2006, that contains VOCs in excess of specific standards. The Clean Air Act Amendments of 1990 establish tighter VOC emission standards for automobiles and trucks and also set minimum requirements for a fee system for VOC emissions [53]. Many states have implemented policies to reduce VOC emissions and impose emissions fees as well. Five states provide financial incentives or other benefits for "clean fuel" with low VOC emissions. Three states require or recommend the use of clean fuel or clean fuel vehicles, and one state has established working groups to study clean vehicle implementation.

Seven states have legislation that specifically require the disclosure of mold on property transactions, and Virginia has model regulation that requires landlords to disclose any "visible evidence" of mold and permits tenants to break a lease within five days if the landlord reports there is visible mold. Four states have policies that address mold in school buildings; of these, California has model regulation that requires school districts to ensure that schools are in "good repair," including no evidence of mold. Nine states created specific mold programs or working groups to assess mold issues in the state, and several states have public education programs.

## Discussion

Our analysis identified few regulations or laws that are specifically intended to protect children. Nonetheless, we identified a number of states that have implemented measures to protect the general public that also are effective in limiting morbidity from asthma and neurodevelopmental disabilities in children. Some of these measures, such as establishment of incentives for lead hazard abatement, reduction of mercury emissions, requirements for schools to phase in green cleaning products, and limits on school bus idling are models that other states and the federal government may wish to consider in the interest of preventing childhood disease.

We limited our analysis to those chemical factors in the environment that have been associated with an increased risk of developmental disability or asthma. There are no doubt other important policies that states have pursued to limit other exposures of concern for children. Our approach did not compare policies regarding polybrominated diphenyl ethers (PBDEs), a type of flame retardants for which there is laboratory evidence to support toxicity of animals, but not in humans. Nine states have banned the use or sale of products containing Penta- or Octa-PBDE, while three states have authorized studies of the hazard posed by use of Deca-PBDE, and Washington has banned its use outright. More human studies are needed to identify whether these legislative interventions may indeed have prevented neurodevelopmental disabilities in children.

We do comment that the absence of a regulation or legislation may underrepresent the effort devoted by states to debating the importance of these issues. Another important caveat to our analysis is that some states may choose not to enact regulation or legislation to limit exposures for contaminants that are not commonly experienced or do not originate in that state. Given that most coal-fired power plants are located in the Northeast and Midwest, efforts to limit mercury emissions from these sources in Southeastern states may not reduce methylmercury contamination of fish eaten by women and children in those states. Nonetheless, we identified many gaps in environmental regulation and legislation that could prevent disease and disability in all states. Lead paint hazards do not obey state boundaries, yet many states have not implemented programs to accelerate process towards eradication of these hazards. Similarly, chloralkali plants are largely located in the Southeast, yet we identified no regulation in those states to limit mercury emissions from these sources.

It is important to note that some state regulations may also result in benefits that cross political boundaries. Regulation of mercury emissions by coal-fired power plant in the Northeast and Midwest prevents mercury pollution from being swept by air currents into the Southeast and may reduce contamination in fish caught elsewhere but sold in the Southeast or other parts of the country. We also applied the same evaluation approach to each state, when in actuality, some policy measures may be more important than others. In agricultural states, legislation preventing take-home exposures from agricultural workers may be more important, while legislation regarding integrated pest management may be more important in more densely populated, urban states. State government officials may wish to view our analysis with this caveat, and researchers may wish to study the impact of "lab experiments" in which states with highly prevalent risks manage them with legislation that reflects local contexts. Our approach also fails to highlight special risks experienced by vulnerable subpopulations of children with genetic predispositions or coexisting exposures that may have additive/synergistic effects.

Further research is also needed to examine the impact of interest groups in shaping how epidemiologic knowledge is translated into policy. The openness and responsiveness of the various state legislatures to adopting model legislation or regulations may be an important determinant of health in its own right. It is beyond the scope of this paper to analyze the historical underpinnings of the legislation and regulation we identified, and this too could be an important area for further research.

Our analysis represents the first published systematic review of children's environmental health legislation at the state level, and we recognize that our approach may not be perfect. However, our validity screen identified only one significant difference between our results and those from other sources – for lead screening. We recognize that the AAP's support and educational efforts to encourage provider screening may be as important if not more so to the prevention of lead exposure, and thus a comparison of states by this metric only provides one perspective on a larger problem. We did not analyze funding of lead screening programs, or effectiveness of state health departments in executing lead abatement where affected children live.

By eliminating public health programs and policies from our analysis, we did eliminate good initiatives which states have implemented to limit children's exposure to hazardous chemicals. Fish advisories are likely to reduce prenatal exposure more immediately [17] than future reduction of mercury emissions of coal-fired power plants. We also comment that we did not analyze efforts by states to perform the necessary inspection and testing to confirm enforcement of environmental legislation and regulation. These are important caveats to judgments that policy makers and the public should consider in weighing the results of our analysis.

Recognizing these limitations, the differences in environmental regulation and legislation we identified are likely to lead to differences in exposure. Reduction of children's exposure to diesel exhaust, a known human carcinogen [54] and exacerbant of asthma [29], is likely to have quantifiable impact on children's health as well. Lead [55] and mercury [37,56] exposure each pose significant economic burdens on America, and reduction of these exposures is likely to result in economic benefits to those states that pursue prevention of environmental hazards. Policy makers may wish to consider the model legislation and regulation we identified for their own states as a proactive investment in the future health of their states' children.

We identified a number of environmental issues for which states exceeded federal standards for environmental protection. While federal regulation and legislation can produce greater uniformity in prevention of childhood disease and disability across states, political and other considerations can prevent enactment of federal law even when scientific evidence abounds to support efforts to limit exposure. Policy makers in the states do not have the luxury of waiting for research to unfold in considering regulation and legislation, and must weigh the scientific evidence for prevention against the many social and political factors that diminish or enhance interest in protecting children's health. Our analysis suggests that many states have many opportunities for improvement, and need not be original in developing new legislation. As Justice Louis Brandeis stated in a 1932 US Supreme Court case, "It is one of the happy incidents of the federal system that a single courageous state may, if its citizens choose, serve as a laboratory; and try novel social and economic experiments without risk to the rest of the country." [57]. The states truly serve that President Thomas Jefferson envisioned for them as policy laboratories for children's environmental health, and provide opportunities for improvement in federal policy as well.

## Conclusion

Few states have regulations or laws that are specifically intended to protect children from environmental hazards but many states that have implemented measures to protect the general public that also are effective in limiting morbidity from asthma and neurodevelopmental disabilities in children. Some of these measures are models that other states and the federal government may wish to consider in the interest of preventing childhood disease.

## Abbreviations

AAP: American Academy of Pediatrics; DPM: Diesel Particulate Matter; LN: Lexis Nexis; PBDE: Polybrominated Diphenyl Ethers; PCB: Polychlorinated Biphenyls; USEPA: US Environmental Protection Agency; VOC: Volatile Organic Compound.

## Competing interests

The authors declare that they have no competing interests.

## Authors' contributions

LZ and ES collected the primary data and performed the preliminary analysis of state environmental regulation and legislation. LT conceived the study and obtained primary funding, and PJL and LT reviewed and refined the analysis of state environmental regulation and legislation.

## References

[B1] USEPA Chemicals-in-commerce information system (Chemical Update System Database) Washington, DC 1998

[B2] Landrigan PJ Trasande L Thorpe LE Gwynn C Lioy PJ D'Alton ME Lipkind HS Swanson J Wadhwa PD Clark EB The National Children's Study: a 21-year prospective study of 100,000 American children Pediatrics 2006 118 2173 2186 10.1542/peds.2006-0360 17079592

[B3] McConnell R Berhane K Gilliland F London SJ Islam T Gauderman WJ Avol E Margolis HG Peters JM Asthma in exercising children exposed to ozone: a cohort study The Lancet 2002 359 386 391 10.1016/S0140-6736(02)07597-9 11844508

[B4] CDC Surveillance for asthma – United States, 1960–1995 MMWR 1998 47 1 28 9580746

[B5] Robison LL Buckley JD Bunin G Assessment of environmental and genetic factors in the etiology of childhood cancers: the Childrens Cancer Group epidemiology program Environmental Health Perspectives 1995 103 Suppl 6 111 8549456 10.2307/3432358 PMC1518916

[B6] Schechter CB Re: Brain and other central nervous system cancers: recent trends in incidence and mortality J Natl Cancer Inst 1999 91 2050 2051 10.1093/jnci/91.23.2050 10580036

[B7] Paulozzi LJ Erickson JD Jackson RJ Hypospadias Trends in Two US Surveillance Systems Pediatrics 1997 100 831 834 10.1542/peds.100.5.831 9346983

[B8] Di Tanna GL Rosano A Mastroiacovo P Prevalence of gastroschisis at birth: retrospective study British Medical Journal 2002 325 1389 1390 12480852 10.1136/bmj.325.7377.1389 PMC138514

[B9] Williams LJ Kucik JE Alverson CJ Olney RS Correa A Epidemiology of gastroschisis in metropolitan Atlanta, 1968 through 2000 Birth Defects Research Part A: Clinical and Molecular Teratology 2005 73 177 183 10.1002/bdra.20114 15744732

[B10] Froehlich TE Lanphear BP Epstein JN Barbaresi WJ Katusic SK Kahn RS Prevalence, Recognition, and Treatment of Attention-Deficit/Hyperactivity Disorder in a National Sample of US Children Archives of Pediatrics and Adolescent Medicine 2007 161 857 10.1001/archpedi.161.9.857 17768285

[B11] Braun JM Kahn RS Froehlich T Auinger P Lanphear BP Exposures to Environmental Toxicants and Attention Deficit Hyperactivity Disorder in US Children Environmental Health Perspectives 2006 114 1904 17185283 10.1289/ehp.9478 PMC1764142

[B12] Grandjean P Landrigan PJ Developmental neurotoxicity of industrial chemicals The Lancet 2006 368 2167 2178 10.1016/S0140-6736(06)69665-7 17174709

[B13] Goldman L Falk H Landrigan PJ Balk SJ Reigart JR Etzel RA Environmental Pediatrics and Its Impact on Government Health Policy Pediatrics 2004 113 1146 15060212

[B14] Grosse SD Matte TD Schwartz J Jackson RJ Economic Gains Resulting from the Reduction in Children's Exposure to Lead in the United States Environmental Health Perspectives 2002 110 563 569 12055046 10.1289/ehp.02110563 PMC1240871

[B15] Whyatt RM Rauh V Barr DB Camann DE Andrews HF Garfinkel R Hoepner LA Diaz D Dietrich J Reyes A Prenatal Insecticide Exposures and Birth Weight and Length among an Urban Minority Cohort Environmental Health Perspectives 2004 112 1125 1132 15238288 10.1289/ehp.6641 PMC1247388

[B16] Friedman MS Powell KE Hutwagner L Graham LRM Teague WG Impact of Changes in Transportation and Commuting Behaviors During the 1996 Summer Olympic Games in Atlanta on Air Quality and Childhood Asthma Am Med Assoc; 2001 285 897 905 10.1001/jama.285.7.897 11180733

[B17] Oken E Kleinman KP Berland WE Simon SR Rich-Edwards JW Gillman MW Decline in Fish Consumption Among Pregnant Women After a National Mercury Advisoryacogjnl 2003 102 346 351 10.1016/S0029-7844(03)00484-8 PMC1989666 12907111

[B18] World Health Organization Children's environmental health indicators http://www.who.int/ceh/indicators/en/(Accessed 28 July 2008).

[B19] America's Children and the Environment (ACE) http://www.epa.gov/economics/children/Accessed 4 March 2008.

[B20] Environmental Health Legislation Database http://www.ncsl.org/programs/environ/envhealth/cehdb.cfm

[B21] Grandjean P Landrigan PJ Developmental neurotoxicity of industrial chemicals The Lancet 2007 368 2167 2178 10.1016/S0140-6736(06)69665-7 17174709

[B22] Etzel RA Balk SJ eds Pediatric Environmental Health 2003 2 Elk Grove Village, IL: American Academy of Pediatrics

[B23] Grandjean P Budtz-Jorgensen E White RF Jorgensen PJ Weihe P Debes F Keding N Methylmercury Exposure Biomarkers as Indicators of Neurotoxicity in Children Aged 7 Years © 1999 by The Johns Hopkins University, School of Hygiene and Public Health 2003 150 301 305 10.1093/oxfordjournals.aje.a010002 10430235

[B24] Weitzman M Gortmaker S Walker DK Sobol A Maternal smoking and childhood asthma Pediatrics 1990 85 505 511 2314963

[B25] Herrmann M King K Weitzman M Prenatal tobacco smoke and postnatal secondhand smoke exposure and child neurodevelopment Current Opinion in Pediatrics 2008 20 184 10.1097/MOP.0b013e3282f56165 18332716

[B26] Laraque D Trasande L Lead poisoning: successes and 21st century challenges Pediatr Rev 2005 26 435 443 16327024

[B27] Jacobson JL Jacobson SW Intellectual Impairment in Children Exposed to Polychlorinated Biphenyls in Utero New England Journal of Medicine 1996 335 783 10.1056/NEJM199609123351104 8703183

[B28] Nakajima S Saijo Y Kato S Sasaki S Uno A Kanagami N Hirakawa H Hori T Tobiishi K Todaka T Effects of Prenatal Exposure to Polychlorinated Biphenyls and Dioxins on Mental and Motor Development in Japanese Children at 6 Months of Age Environmental Health Perspectives 2006 114 773 778 16675436 10.1289/ehp.8614 PMC1459935

[B29] Trasande L Thurston GD The role of air pollution in asthma and other pediatric morbidities J Allergy Clin Immunol 2005 115 689 699 10.1016/j.jaci.2005.01.056 15805986

[B30] Kattan M Stearns SC Crain EF Stout JW Gergen PJ Evans R Iii Visness CM Gruchalla RS Morgan WJ O'Connor GT Cost-effectiveness of a home-based environmental intervention for inner-city children with asthma Journal of Allergy and Clinical Immunology 2005 116 1058 1063 10.1016/j.jaci.2005.07.032 16275376

[B31] Gent JF Ren P Belanger K Triche E Bracken MB Holford TR Leaderer BP Levels of household mold associated with respiratory symptoms in the first year of life in a cohort at risk for asthma Environmental Health Perspectives 2002 110 12 A781 12460818 10.1289/ehp.021100781 PMC1241132

[B32] Rumchev K Spickett J Bulsara M Phillips M Stick S Association of domestic exposure to volatile organic compounds with asthma in young children Thorax 2004 59 746 15333849 10.1136/thx.2003.013680 PMC1747137

[B33] Centers for Disease Control and Prevention Best Practices for Comprehensive Tobacco Control Programs-2007 2008 http://www.cdc.gov/tobacco/tobacco_control_programs/stateandcommunity/best_practices/

[B34] Centers for Disease Control and Prevention Best Practices of Youth Violence Prevention: A Sourcebook for Community Action 2008 http://www.helpingamericasyouth.gov/exhibithall/CDC%20-%20Best%20Practices%20of%20Violence%20Prevention.pdf

[B35] Parker SK Schwartz B Todd J Pickering LK Thimerosal-Containing Vaccines and Autistic Spectrum Disorder: A Critical Review of Published Original Data Pediatrics 2004 114 3 793 804 10.1542/peds.2004-0434 15342856

[B36] U.S. Environmental Protection Agency Technology Transfer Network (TTN), Clearinghouse for Inventories and Emissions Factors. 2003 National Emissions Inventories for Hazardous Air Pollutants 1999 http://www.epa.gov/ttn/chief Version 3, July 2003. Accessed May 18, 2004.

[B37] Trasande L Landrigan PJ Schechter C Public health and economic consequences of methyl mercury toxicity to the developing brain Environ Health Perspect 2005 113 5 590 596 15866768 10.1289/ehp.7743 PMC1257552

[B38] Curl CL Fenske RA Kissel JC Shirai JH Moate TF Griffith W Coronado G Thompson B Evaluation of take-home organophosphorus pesticide exposure among agricultural workers and their children Environmental Health Perspectives 2002 110 A787 12460819 10.1289/ehp.021100787 PMC1241133

[B39] Lu C Toepel K Irish R Fenske RA Ban DB Bravo R Organic Diets Significantly Lower Children's Dietary Exposure to Organophosphorus Pesticides Environmental Health Perspectives 2006 114 260 263 16451864 10.1289/ehp.8418 PMC1367841

[B40] Brenner BL Markowitz S Rivera M Romero H Weeks M Sanchez E Deych E Garg A Godbold J Wolff MS Integrated pest management in an urban community: a successful partnership for prevention Environmental Health Perspectives 2003 111 1649 1653 14527845 10.1289/ehp.6069 PMC1241688

[B41] Wasserman GA Liu X Parvez F Ahsan H Factor-Litvak P van Geen A Slavkovich V Lolacono NJ Cheng Z Hussain I Water Arsenic Exposure and Children's Intellectual Function in Araihazar, Bangladesh Environmental Health Perspectives 2004 112 1329 15345348 10.1289/ehp.6964 PMC1247525

[B42] Kwon E Zhang H Wang Z Jhangri GS Lu X Fok N Gabos S Li XF Le XC Arsenic on the Hands of Children after Playing in Playgrounds Environmental Health Perspectives 2004 112 1375 1380 15471728 10.1289/ehp.7197 PMC1247563

[B43] Townsend T Solo-Gabriele H Tolaymat T Stook K Hosein N Chromium, Copper, and Arsenic Concentrations in Soil Underneath CCA-Treated Wood Structures Soil and Sediment Contamination 2003 12 779 798 10.1080/714037715

[B44] Committee on Environmental H Ambient Air Pollution: Health Hazards to Children Pediatrics 2004 114 1699 1707 10.1542/peds.2004-2166 15574638

[B45] Adgate JL Church TR Ryan AD Ramachandran G Fredrickson AL Stock TH Morandi MT Sexton K Outdoor, Indoor, and Personal Exposure to VOCs in Children Environmental Health Perspectives 2004 112 14 1386 15471730 10.1289/ehp.7107 PMC1247565

[B46] Seltzer JM Fedoruk MJ Health Effects of Mold in Children The Pediatric Clinics of North America 2007 54 309 333 10.1016/j.pcl.2007.02.001 17448362

[B47] Lexis Nexis Academic: State Codes, Constitutions, Court Rules & Advance Legislative Service http://www.lexisnexis.com/us/lnacademic/search/loadForm.do?formID=AC07STFedStCodesSrch&random=0.6770431099682575

[B48] State and Local Programs, Childhood Lead Poisoning Prevention Program http://www.cdc.gov/nceh/lead/grants/contacts/CLPPP%20Map.htm

[B49] State of Tobacco Control 2006: National Grades http://lungaction.org/reports/rank-states06.html

[B50] National Conference of State Legislators (NCSL) Environmental Health Database 1998 http://www.ncsl.org/programs/environ/envhealth/toxics.htm

[B51] National Research Council (Subcommittee on Arsenic in Drinking Water) Arsenic in Drinking Water 1999 Washington, DC: National Academy Press

[B52] Response to Requests to Cancel Certain Chromated Copper Arsenate (CCA) Wood Preservative Products and Amendments to Terminate Certain Uses of other CCA Products http://www.epa.gov/EPA-PEST/2003/April/Day-09/p8372.htm

[B53] Clean Air Act http://www.epa.gov/air/caa/

[B54] International Agency for Research on Cancer (IARC) DIESEL AND GASOLINE ENGINE EXHAUSTS IARC Monographs on the Evaluation of Carcinogenic Risks to Humans 1989 46 PMC7681285 2483418

[B55] Landrigan PJ Schechter CB Lipton JM Fahs MC Schwartz J Environmental Pollutants and Disease in American Children: Estimates of Morbidity, Mortality, and Costs for Lead Poisoning, Asthma, Cancer, and Developmental Disabilities Environmental Health Perspectives 2002 110 721 728 12117650 10.1289/ehp.02110721 PMC1240919

[B56] Trasande L Schechter CB Haynes KA Landrigan PJ Mental retardation and prenatal methylmercury toxicity Am J Ind Med 2006 49 153 158 10.1002/ajim.20268 16470549

[B57] Bernholz P Vaubel R Political Competition and Economic Regulation: Routledge 2007

